# Effects of Wall Shear Stress on Unsteady MHD Conjugate Flow in a Porous Medium with Ramped Wall Temperature

**DOI:** 10.1371/journal.pone.0090280

**Published:** 2014-03-12

**Authors:** Arshad Khan, Ilyas Khan, Farhad Ali, Sami ulhaq, Sharidan Shafie

**Affiliations:** Department of Mathematical Sciences, Faculty of Science, Universiti Teknologi Malaysia, Skudai, Malaysia; College of Engineering, Majmaah University, Majmaah, Saudi Arabia; City University of Science and Information Technology, Peshawar, Pakistan; University of Zurich, Switzerland

## Abstract

This study investigates the effects of an arbitrary wall shear stress on unsteady magnetohydrodynamic (MHD) flow of a Newtonian fluid with conjugate effects of heat and mass transfer. The fluid is considered in a porous medium over a vertical plate with ramped temperature. The influence of thermal radiation in the energy equations is also considered. The coupled partial differential equations governing the flow are solved by using the Laplace transform technique. Exact solutions for velocity and temperature in case of both ramped and constant wall temperature as well as for concentration are obtained. It is found that velocity solutions are more general and can produce a huge number of exact solutions correlative to various fluid motions. Graphical results are provided for various embedded flow parameters and discussed in details.

## Introduction

In many practical situations such as condensation, evaporation and chemical reactions the heat transfer process is always accompanied by the mass transfer process. Perhaps, it is due to the fact that the study of combined heat and mass transfer is helpful in better understanding of a number of technical transfer processes. Besides, free convection flows with conjugate effects of heat and mass transfer past a vertical plate have been studied extensively in the literature due to its engineering and industrial applications in food processing and polymer production, fiber and granular insulation and geothermal systems [1]–[3]. Some recent attempts in this area of research are given in [4]–[9]. On the other hand, considerable interest has been developed in the study of interaction between magnetic field and the flow of electrically conducting fluids in a porous medium due to its applications in modern technology [10]. Toki et al. [11] have studied the unsteady free convection flows of incompressible viscous fluid near a porous infinite plate with arbitrary time dependent heating plate. The effects of chemical reaction in two dimensional steady free convection flow of an electrically conducting viscous fluid through a porous medium bounded by vertical surface with slip flow region has been studied by Senapati1 et al. [12]. Khan et al. [13] analyzed the effects of radiation and thermal diffusion on MHD free convection flow of an incompressible viscous fluid near an oscillating plate embedded in a porous medium.

The influence of magnetic field on the other hand is observed in several natural and human-made flows. Magnetic fields are commonly applied in industry to pump, heat, levitate and stir liquid metals. There is the terrestrial magnetic field which is maintained by fluid flow in the earth's core, the solar magnetic field which originates sunspots and solar flares, and the galactic magnetic field which is thought to control the configuration of stars from interstellar clouds [14]. Recently, considerable attention has been focused on applications of MHD and heat transfer such as metallurgical processing, MHD generators and geothermal energy extraction. The phenomenon concerning heat and mass transfer with MHD flow is important due to its numerous applications in science and technology. The particular applications are found in buoyancy induced flows in the atmosphere, in bodies of water and quasi-solid bodies such as earth. Therefore, heat and mass transfer with MHD flow has been a subject of concern of several researchers including Hayat et al. [15], Jha and Apere [16] and Fetecau et al. [17].

Furthermore, it is found from the literature that several investigations on free convection flows are available with different thermal conditions at the bounding plate which are continuous and well-defined at the wall. However, most of the practical problems appear with non-uniform or arbitrary conditions at the wall. To study such problems, it is useful to investigate them under step change in wall temperature. The physical implication of this idea can be found in the fabrication of thin-film photovoltaic devices where ramped wall temperatures may be employed to achieve a specific finish of the system [18]. According to [19], periodic temperature step changes are also important in building heat transfer applications such as in air conditioning, where the conventional assumption of periodic outdoor conditions may lead to considerable errors in the case of a significant temporary deviation of the temperature from periodicity. Keeping this in view, several authors have studied free convection flow past a vertical plate with step discontinuities in the surface temperature. However, here we are only highlighting some recent and important contributions [20]–[25].

On the other hand, the motion of the fluid past an infinite plate is of great interest for academic research due to its various practical applications. Of course such motion can be induced as a results of several effects including motions due to boundaries and applications of the wall shear stress. Exact solutions of the problems with shear stress on the bounding plate are quite complicated and therefore, very few studies are available in the literature. Such studies are even scarce with combined effects of heat and mass transfer. Navier [26] had proposed a slip boundary condition where the slip velocity depends linearly on the shear stress. Generally, the slip velocity strongly depends on the shear stress and mostly governing equations for slip are developed under this assumption. The slip that appears at the wall has led to the study of an interesting class of problems in which the shear stress is given on the solid boundary. Having such motivation in mind, Fetecau et al. [28] investigated free convection flow near a vertical plate that applies arbitrary shear stress to the fluid when the thermal radiation and porosity effects are taken into consideration. However, so far no study has been reported in the literature which focuses on the conjugate free convection flow with ramped wall temperature under the arbitrary shear stress condition. Even such studies are not available for viscous fluids.

Therefore, the aim of the present investigation is to provide exact solutions for MHD conjugate flow of a Newtonian fluid past an infinite plate that applies arbitrary shear stress to the fluid. More exactly, we consider the vertical plate situated in the 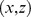 plane of a Cartesian coordinate system 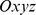, the domain of the flow is the porous half-space 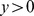 and the arbitrary shear stress on the vertical plate is given by 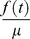, where 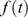 is an arbitrary function and 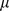 is the viscosity. Closed form solutions of the initial and boundary value problems that govern the flow are obtained by means of the integral transform method. Some special cases are extracted from the general solutions together with some limiting solutions in the literature. The results for velocity, temperature and concentration profiles are plotted graphically and discussed for the embedded flow parameters.

## Mathematical Formulation

**Figure 1:**
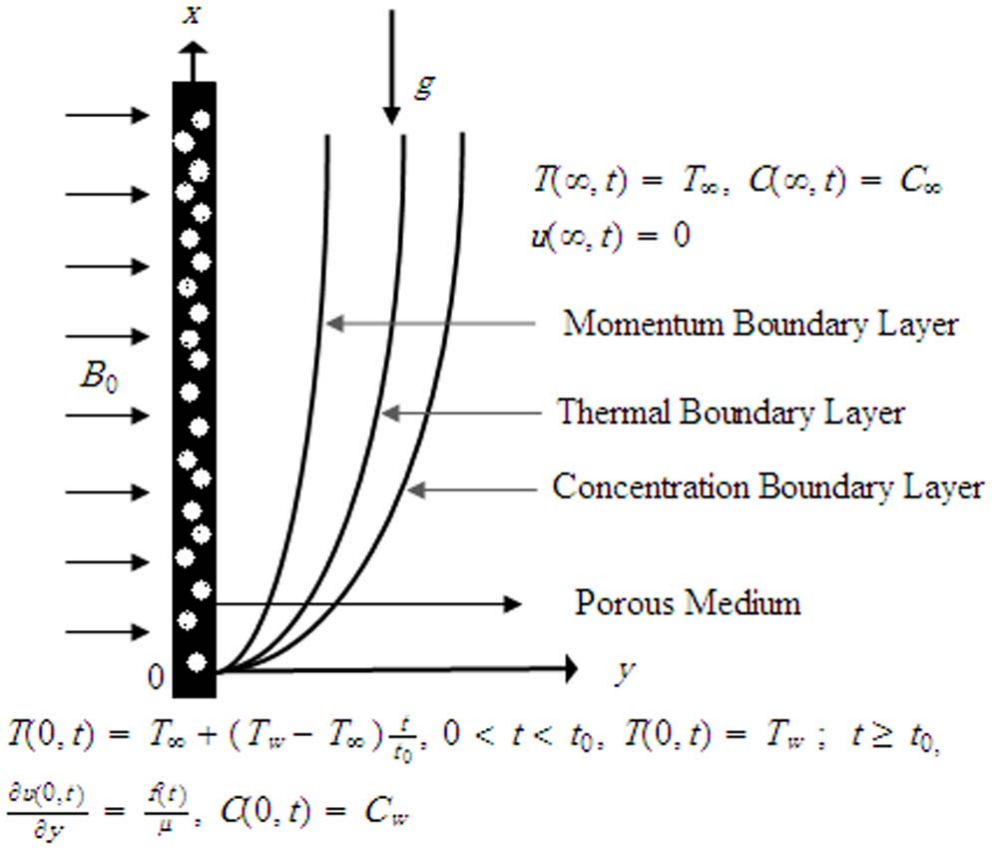
Physical configuration of the problem.

Let us consider the unsteady free convection flow of an incompressible viscous fluid over an infinite vertical plate embedded in a porous medium. The physical configuration of the problem is shown in Fig. 1. The 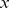-axis is taken along the plate and the 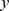-axis is taken normal to it. Initially, both the plate and fluid are at stationary conditions with the constant temperature 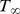 and concentration 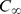. After time 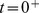, the plate applies a time dependent shear stress 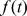 to the fluid along the 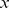-axis. Meanwhile, the temperature of the plate is raised or lowered to 
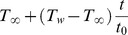
 when 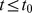, and thereafter, for 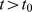, is maintained at constant temperature 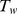 and concentration is raised to 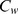. The radiation terms is also considered in the energy equation. However, the radiative heat flux is considered negligible in 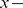
direction compare to 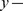
direction. We assume that the flow is laminar and the fluid is grey absorbing-emitting radiation but no scattering medium. In addition to that we asume that the fluid is electrically conducting. Therefore, we use the following Maxwell equations
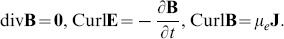
(1)


In the above equations, 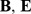 and 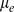 are the magnetic field, electric field intensity and the magnetic permeability of the fluid, respectively. By using Ohm's law, the current density 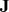 is given as
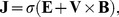
(2)where 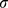 is the electrical conductivity of the fluid. Further we make the following assumptions:

- The quantities 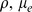 and 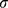 are all constants throughout the flow field.
- The magnetic field 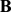 is perpendicular to the velocity field 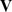.
- The induced magnetic field 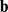 is negligible compared with the imposed magnetic field 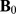.
- The magnetic Reynolds number is small.
- The electric field is zero.

In view of above assumptions, the electromagnetic body force takes the linearized form [15]

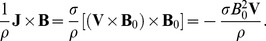
(3)


Using Boussinesq's approximation and neglecting the viscous dissipation, the equations governing the flow are given by [2], [32]

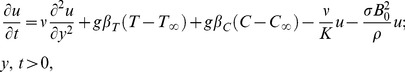
(4)

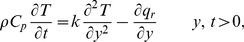
(5)

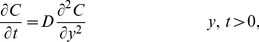
(6)where 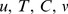, 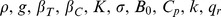, and 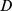 are the velocity of the fluid in 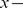
direction, its temperature and concentration, the kinematic viscosity, the constant density, the gravitational acceleration, the heat transfer coefficient, the mass transfer coefficient, the permeability of the porous medium, the electric conductivity of the fluid, the applied magnetic field, the heat capacity at constant pressure, the thermal conductivity, the radiative heat flux and mass diffusivity.

The corresponding initial and boundary conditions are
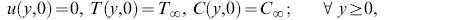


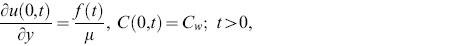


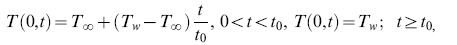


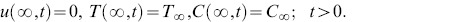
(7)


The radiation heat flux under Rosseland approximation for optically thick fluid [8], [9], [29], [30], [31] is given by
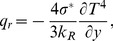
(8)where 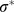 and 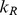 are the Stefan-Boltzmann constant and the mean spectral absorption coefficient respectively. It is supposed that the temperature difference within the flow are sufficiently small, then Eq. (8) can be linearized by expanding 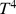 into Taylor series about 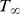, and neglecting higher order terms, we find that
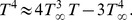
(9)


Substituting Eq. (9) into Eq. (8) and then putting the obtained result in Eq. (5), we get
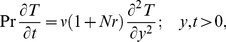
(10)where 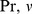 and 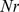 are defined by
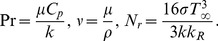
(11)


By introducing the following dimensionless variables
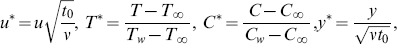


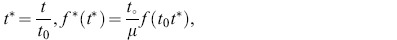
(12)into Eqs. (4), (6) and (10) and dropping out the star notations, we get
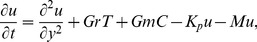
(13)

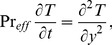
(14)

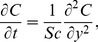
(15)where 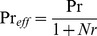 is the effective Prandtl number [29]; Eq. (10)

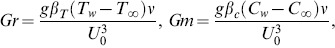


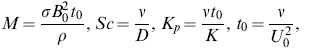
are the Grashof number, modified Grashof number, magnetic parameter, Schmidt number, the inverse permeability parameter for the porous medium and the characteristic time respectively.

The corresponding dimensionless initial and boundary conditions are
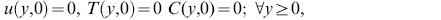


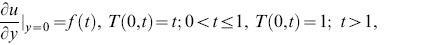
(16)

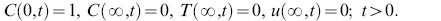



## Solution of the Problem

In order to solve Eqs. (13)–(15) under conditions (16), we use the Laplace transform technique and get the following differential equations
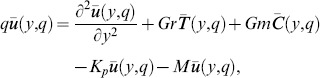
(17)

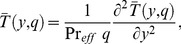
(18)

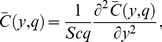
(19)with boundary conditions
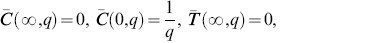


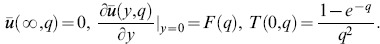
(20)


Solving Eq. (18) in view of Eq. (20), we get
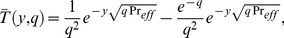
(21)which upon inverse Laplace transform gives
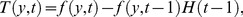
(22)where
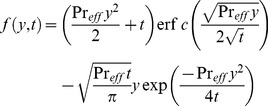
(23)and
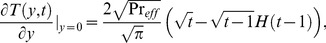
(24)is the corresponding heat transfer rate also known as Nusselt number. Here 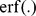 and 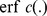 denote the error function and complementary error function of Gauss [28].

Solution of Eq. (19) using boundary conditions from Eq. (20) yields
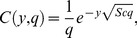
(25)which upon inverse Laplace transform gives
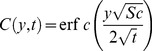
(26)and
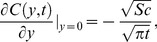
(27)is the corresponding mass transfer rate also known as Sherwood number.

The solution of Eq. (17) under boundary conditions (20) results
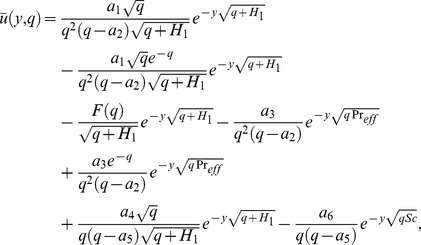
(28) which upon inverse Laplace transform results
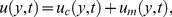
(29)where
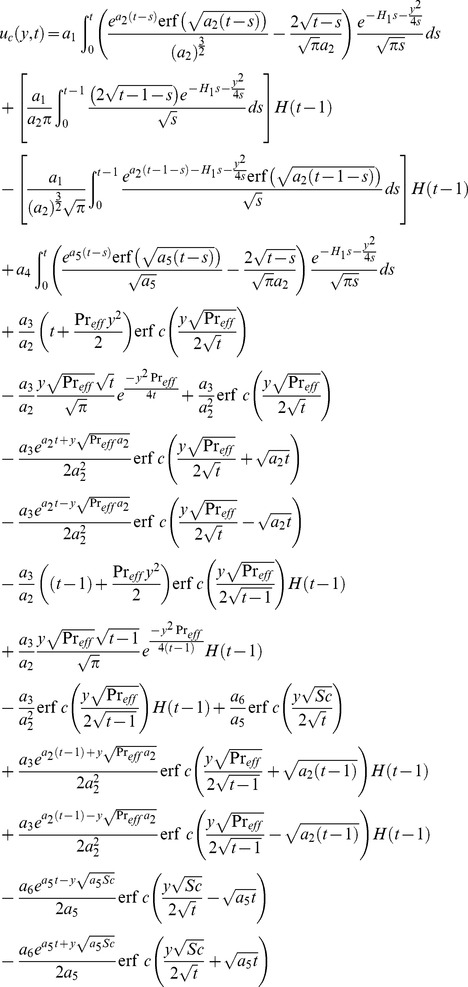
(30)and
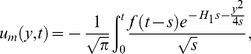
(31)correspond to the convective and mechanical parts of velocity.

It is noted from Eqs. (22) and (30) that 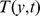 is valid for all positive values of 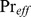 while the 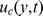 is not valid for 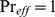. Therefore, to get 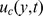 when the effective Prandtl number is not equal to one, we make 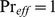 into Eq. (14), use a similar procedure as discussed above, and obtain
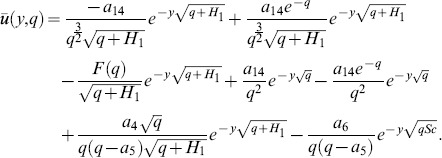
(32)


By taking inverse Laplace transform we find that
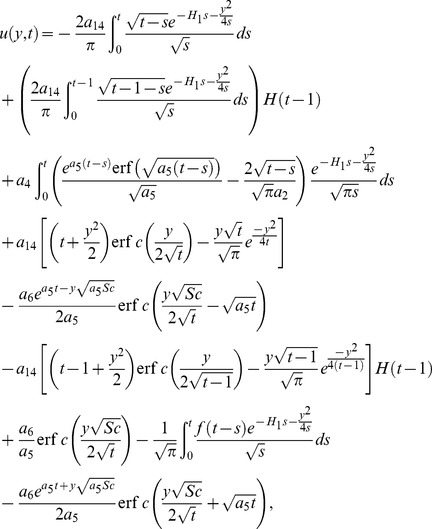
(33)where
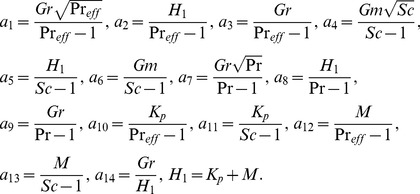
(34)


## Plate with Constant Temperature

Equations (22) and (29) give analytical expressions for the temperature and velocity near a vertical plate with ramped temperature. In order to highlight the effect of the ramped temperature distribution of the boundary on the flow, it is important to compare such a flow with the one near a plate with constant temperature. It can be shown that the temperature, rate of heat transfer and velocity for the flow near an isothermal plate are
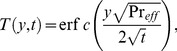
(35)

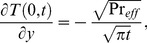
(36)

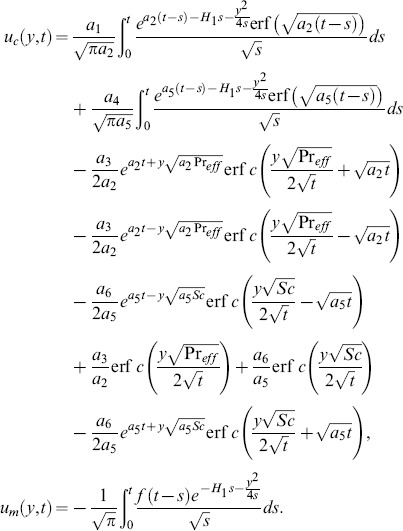
(37)


As previously, Eq. (37) is not valid for 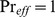. Therefore we calculate separately solution for velocity by taking 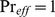 into Eq. (14) and finally get
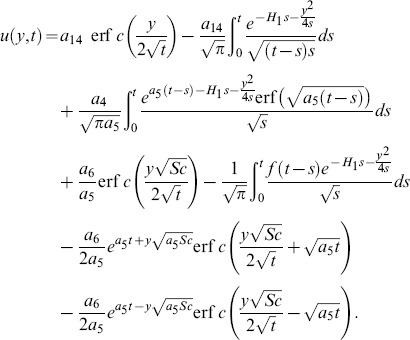
(38)


## Limiting Cases

In this section we discuss few limiting cases of our general solutions.

### 5.1 Solution in the absence of porous effects for ramped and constant wall temperature (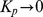)



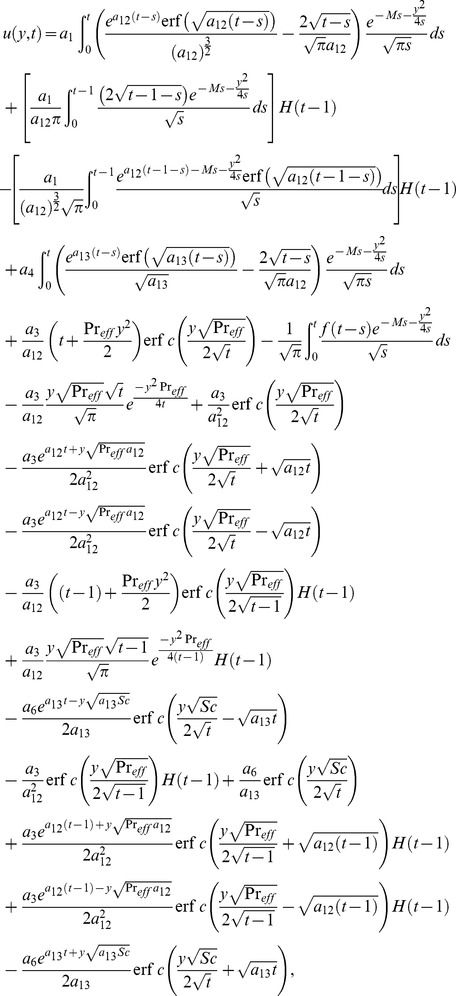
(39)

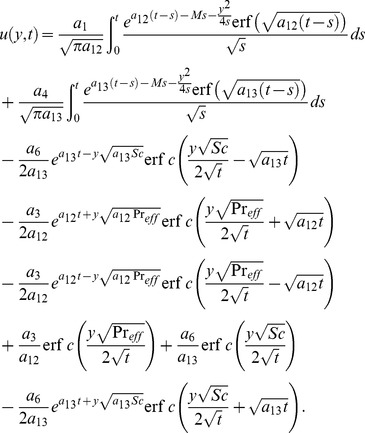
(40)


### 5.2 Solution in the absence of thermal radiation (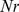 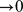)

In the absence of thermal radiation, the corresponding solutions for ramped and constant wall temperature are directly obtained from the general solutions (22), (24), (29) and (35)–(37) by taking 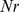 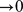 and replacing 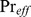 by 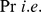


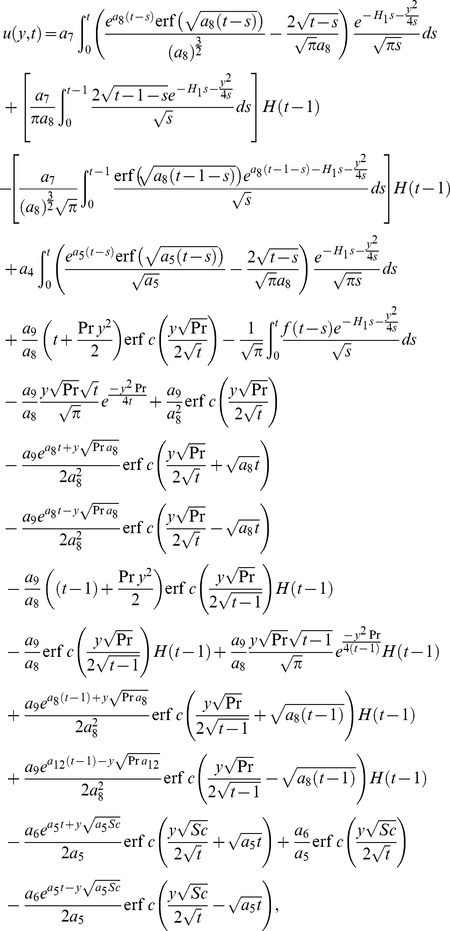
(41)

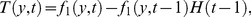
(42)where
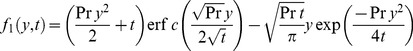
(43)and
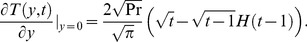
(44)

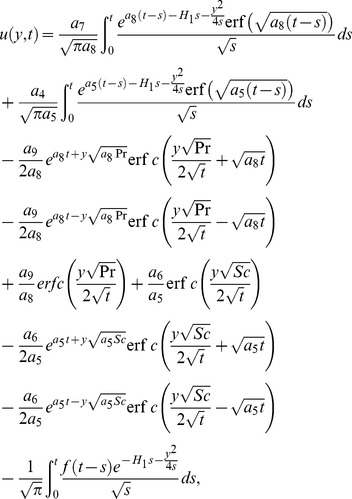
(45)

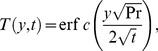
(46)

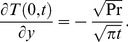
(47)


### 5.3 Solutions in the absence of free convection

Let us assume that the flow is caused only due to bounding plate and the corresponding buoyancy forces are zero equivalently it shows the absence of free convection due to the differences in temperature and mass gradients i.e. the terms 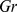 and 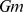 are zero. This shows that the convective parts of velocities are zero in both cases of ramped wall and constant temperature and the flow is only governed by the mechanical part of velocities given by Eqs. (31) and (37).

### 5.4 Solutions in the absence of mechanical effects

In this case we assume that the infinite plate is in static position at every time i.e. the function 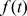 is zero for all values of 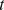 and the mechanical parts for both ramped and constant wall temperature are equivalently zero. In such a situation, the motion in the fluid is induced only due to the free convection which causes due to the buoyancy forces. Therefore, the velocities of the fluid in both cases of ramped and constant wall temperature are only represented by their convective parts given by Eqs. (30) and (37).

### 5.5 Solution in the absence of magnetic parameter (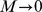)

As it is clear from Eqs. (22) and (26) that the temperature and concentration distributions are not effected by the magnetic parameter 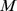, and the velocities with 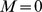 for both ramped and constant wall temperature are given by
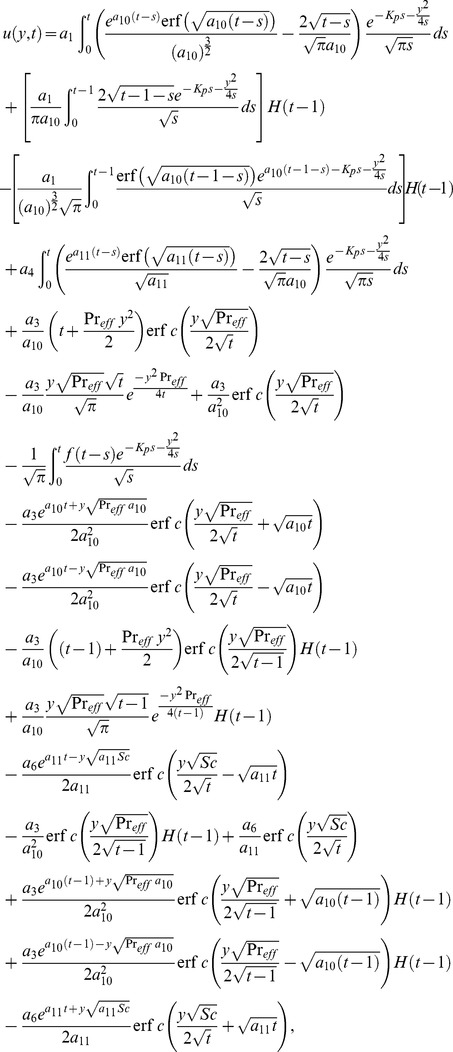
(48)

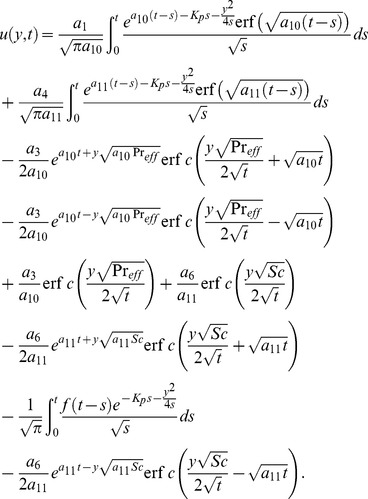
(49)


## Special Cases

As we noted that the solutions for velocity obtained in Section 3, are more general. Therefore, we want to discuss some special cases of the present solutions together with some limiting solutions in order to know more about the physical insight of the problem. Hence, we discuss the following important special cases in the case of ramped wall temperature whose technical relevance is well-known in the literature. Similarly we can discuss some special cases of constant wall temperature solutions.

### 6.1 Case-I: 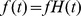


In this first case we take the arbitrary function 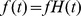, where 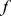 is a dimensionless constant and 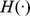 denotes the unit step function. After time 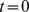, the infinite vertical plate applies a constant shear stress to the fluid. The convective part of the velocity remains unchanged while the mechanical part takes the following form
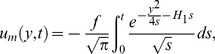
(50)equivalently
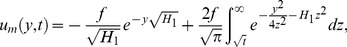
(51)for 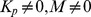. Moreover, if we take 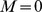, Eq. (50) reduces to the form
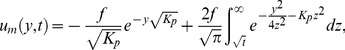
(52)which is equivalent to [28]; Eq. (28) with the correction of 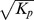.

Furthermore, in the absence of both 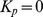 and 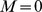, Eq. (50) is identical with [27]; Eq. (23)

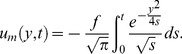
(53)


### 6.2 Case-II: 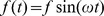


In the second case, we take the arbitrary function of the form 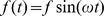 in which the plate applies an oscillating shear stress to the fluid. Here 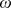 denotes the dimensionless frequency of the shear stress. As previously, the convective part of velocity remains the same whereas the mechanical part takes the form
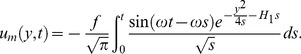
(54)


It can be further written as a sum of the steady-state and transient solutions
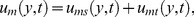
(55)where
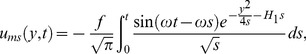
(56)

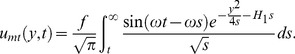
(57)


By taking 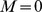, the steady-state component reduces to [28]; Eq. (35)

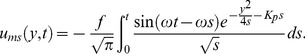
(58)


In addition when 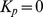, physically it corresponds to the absence of porous effects and Eq. (58) results in
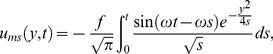
(59)which can be written in simplified form as
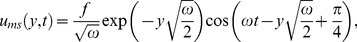
(60)equivalent to [27]; Eq. (33).

### 6.3 Case-III: 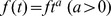


In the final case, we take 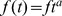, in which the plate applies an accelerating shear stress to the fluid where the mechanical part takes the following form
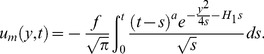
(61)


The corresponding solution for 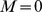, namely
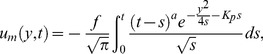
(62)is identical with [28]; Eq. (32).

Additionally, if we take 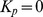, Eq. (62) yields
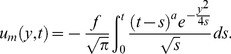
(63)


## Results and Discussion

**Figure 2:**
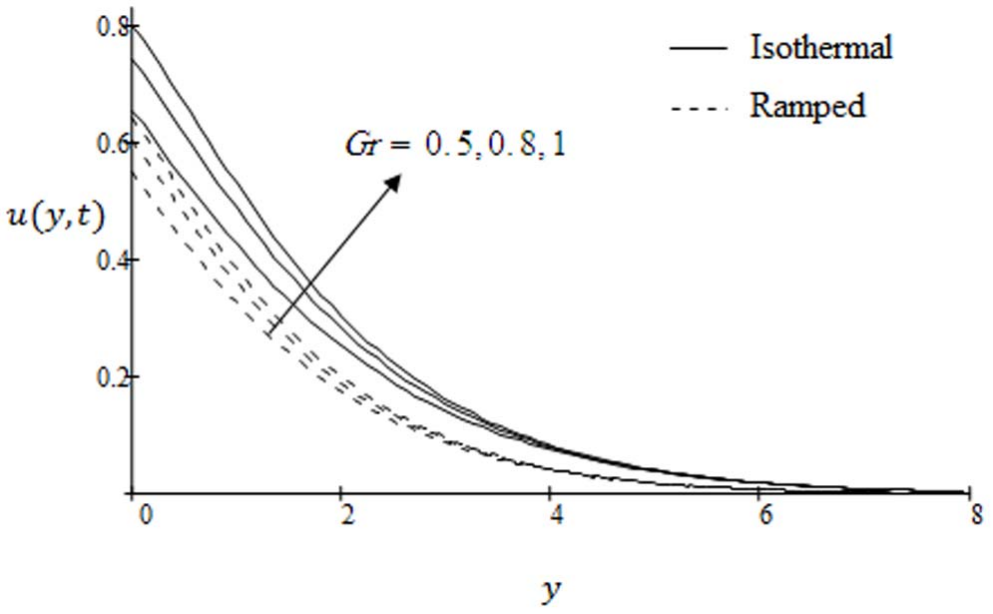
Velocity profiles for different values of 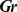 when the plate applies a constant shear stress 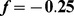.

**Figure 3:**
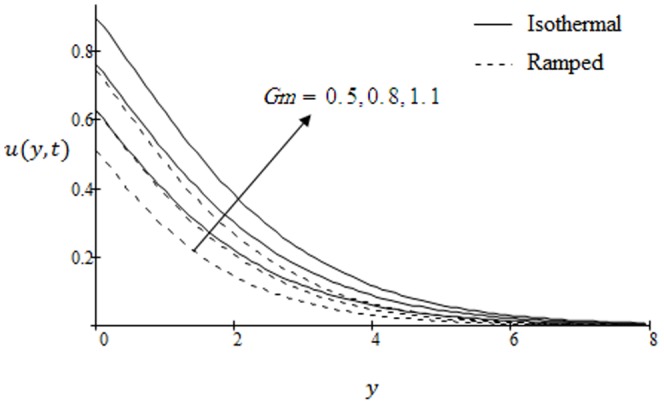
Velocity profiles for different values of 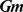 when the plate applies a constant shear stress 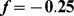.

**Figure 4:**
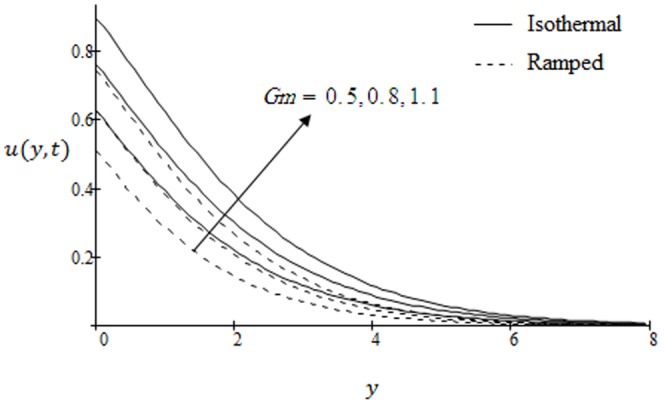
Velocity profiles for different values of 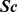 when the plate applies a constant shear stress 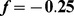.

**Figure 5:**
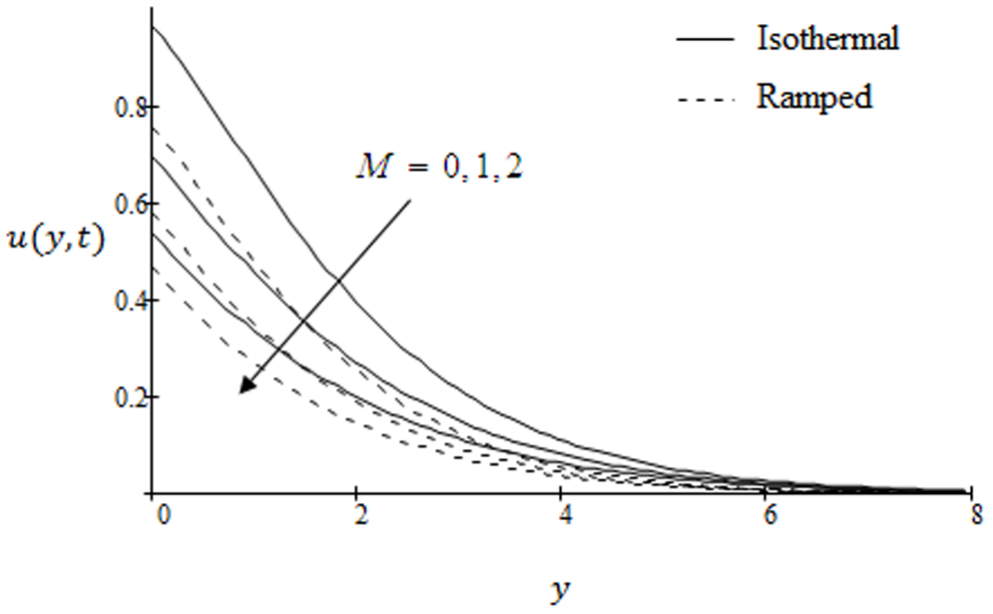
Velocity profiles for different values of 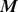 when the plate applies a constant shear stress 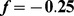.

**Figure 6:**
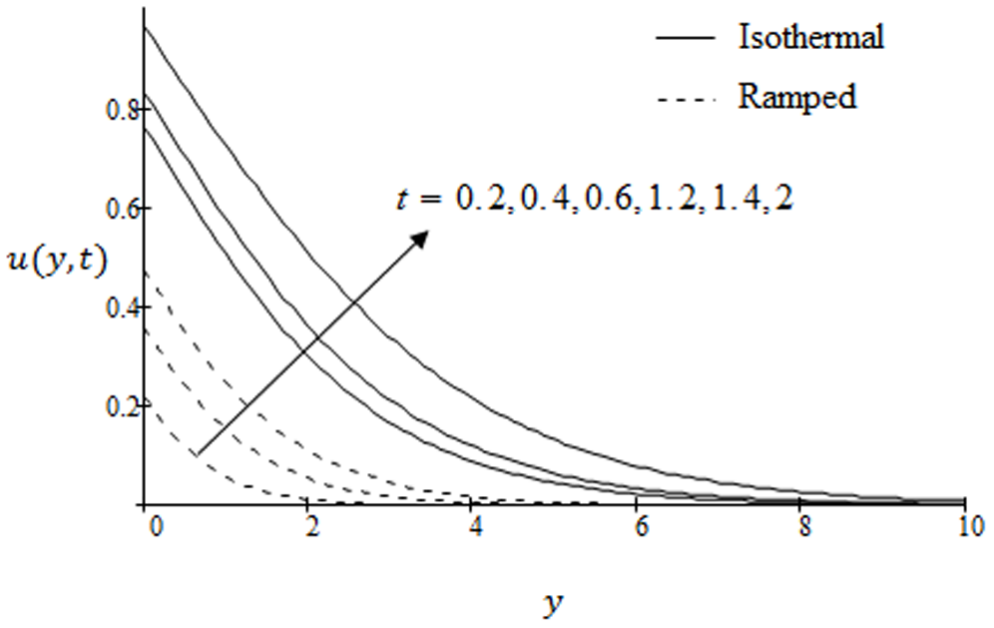
Velocity profiles for different values of 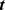 when the plate applies a constant shear stress 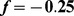.

**Figure 7:**
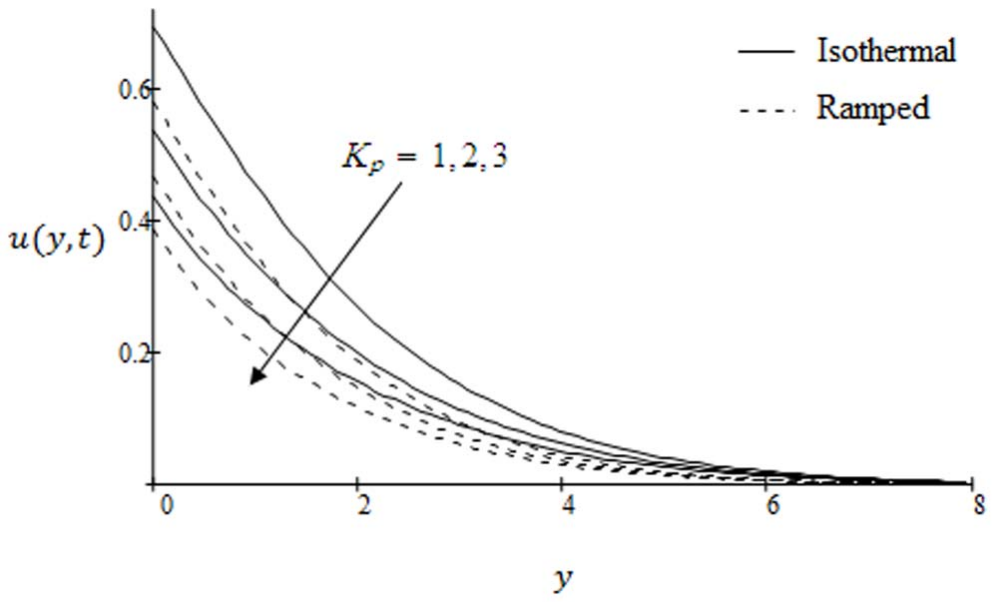
Velocity profiles for different values of 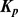 when the plate applies a constant shear stress 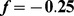.

**Figure 8:**
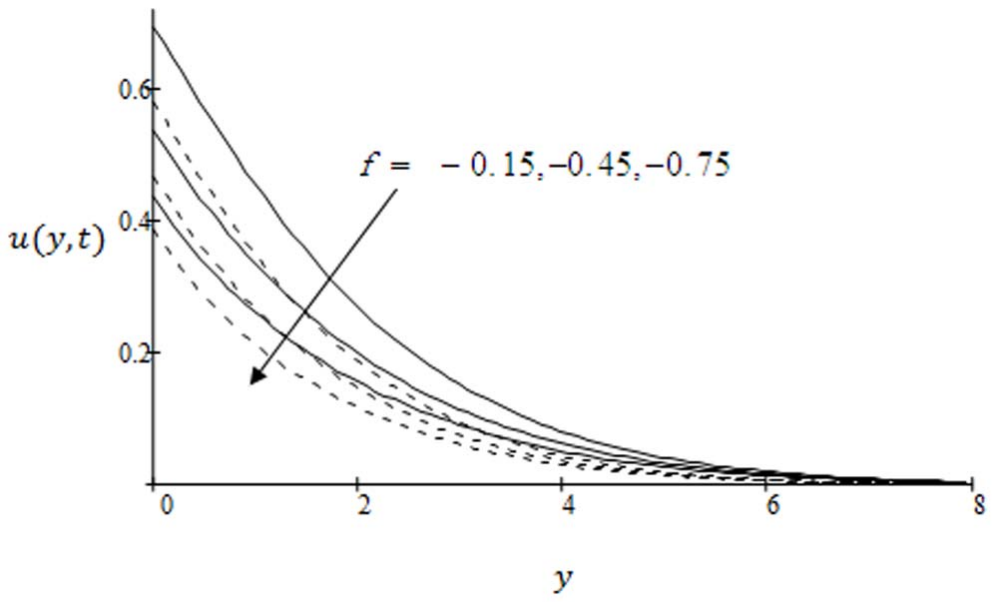
Velocity profiles for different values of constant shear stress 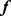.

**Figure 9:**
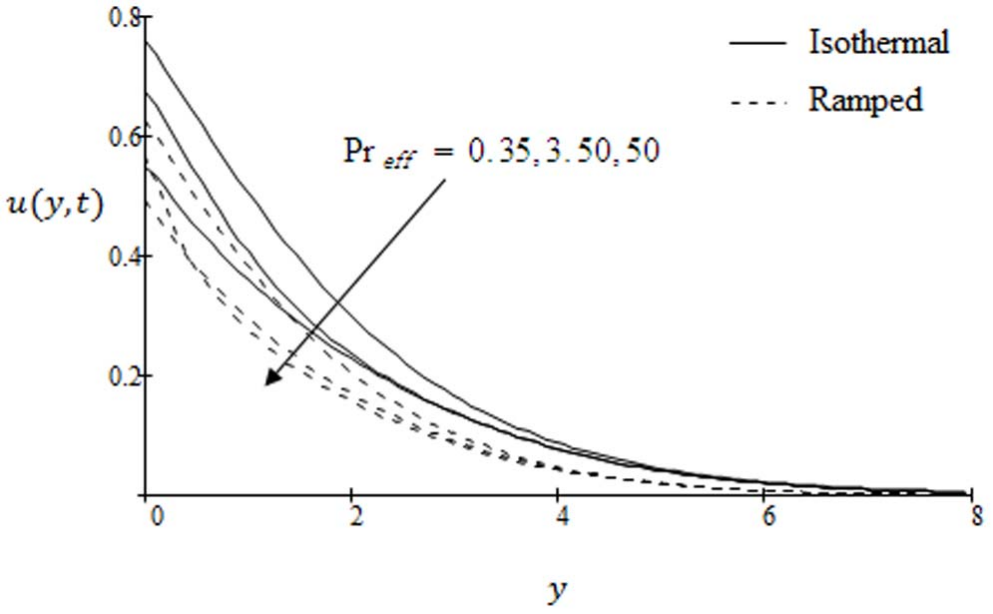
Velocity profiles for different values of 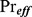 when the plate applies a constant shear stress 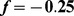.

**Figure 10:**
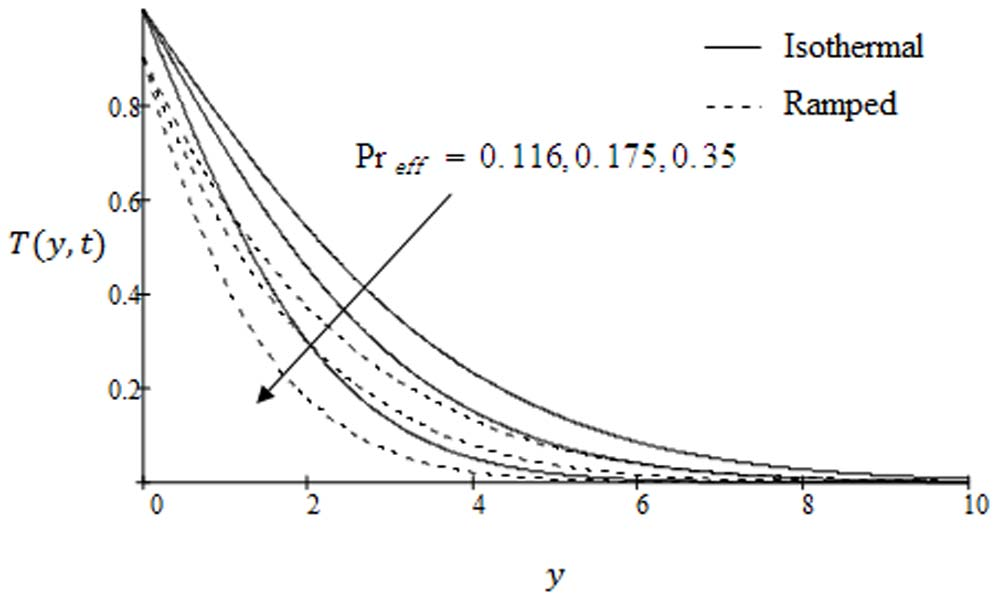
Temperature profile for different values of 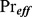.

**Figure 11:**
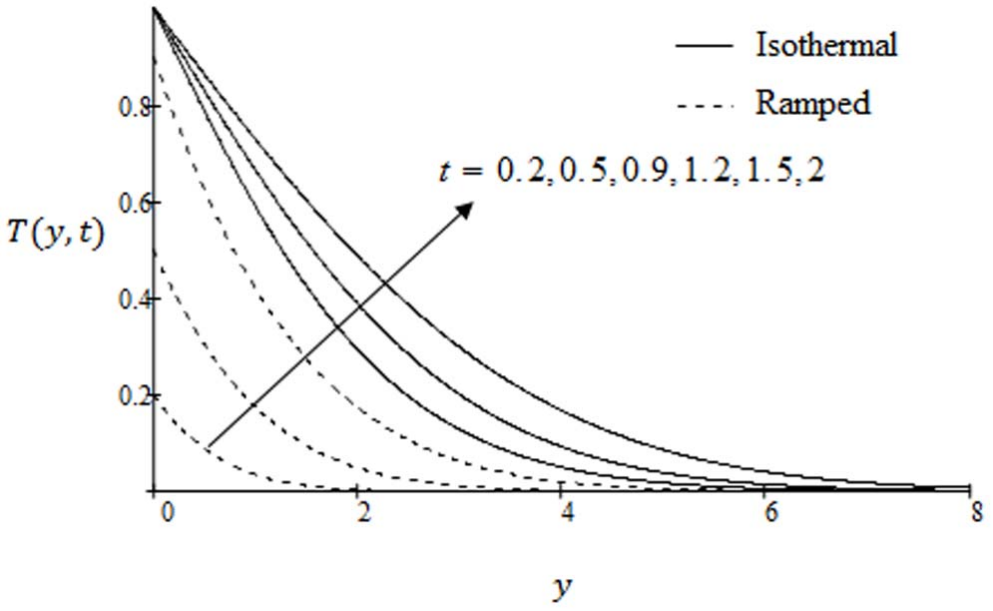
Temperature profiles for different values of 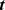.

**Figure 12:**
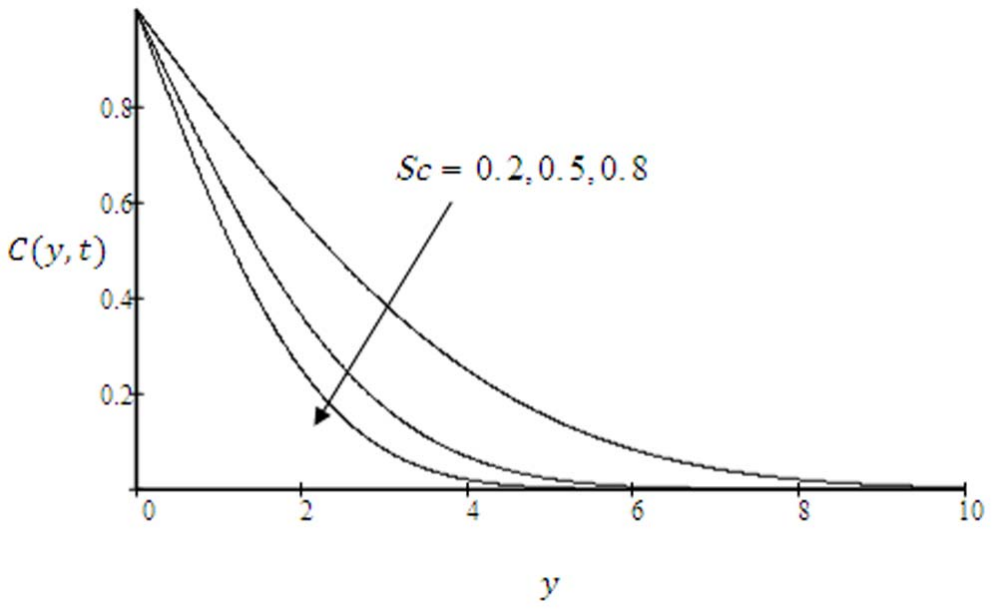
Concentration profiles for 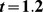 and different values of 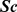.

**Figure 13:**
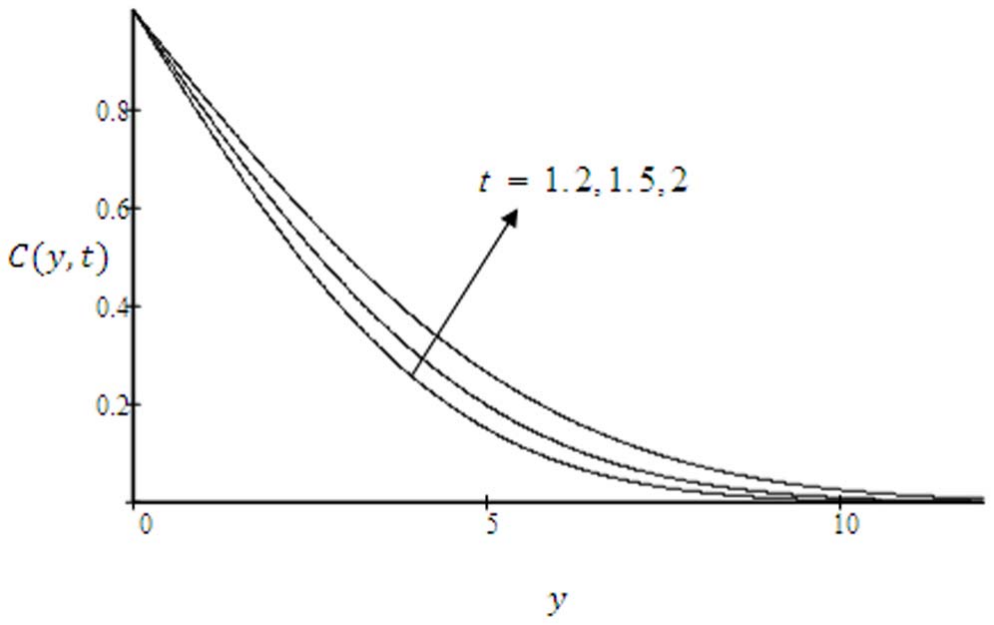
Concentration profiles for 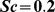 and different values of 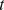.

In order to understand the physical aspects of the problem, the numerical results for velocity, temperature and concentration are computed and plotted for various parameters of interest such as magnetic parameter 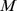, porosity parameter 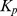, effective Prandtl number 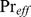, Grashof number 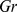, modified Grashof number 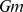, dimensionless time 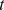, Schmidt number 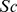 and shear stress 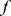. The graphs for velocity are shown in Figs. 2–9 where 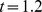 corresponds to isothermal velocity and 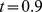 is for ramped velocity. Figs. 10 and 11 are plotted to show the temperature variations for two types of boundary conditions namely ramped and constant wall temperatures. Furthermore, Figs. 12 and 13 are displayed to show variations in fluid concentration. Fig. 2 illustrate the influence of Grashof number 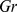 on the velocity. It is observed that velocity increases with increasing 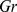. This implies that thermal buoyancy force tends to accelerate velocity for both ramped temperature and isothermal plates. In Fig. 3 the velocity profiles for different values modified Grashof number 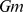 are shown. It is found that velocity increases on increasing 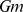 for both ramped temperature and isothermal plate. Further, it can be observed that the velocity and boundary layer thickness decrease along 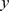 with increasing distance from the the leading edge. Moreover, we observed that the amplitude of velocity in case of isothermal plate is greater and converges slowly as compare to ramped velocity. In Fig. 4 the velocity profiles are shown for different values of Schmidt number 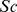. It is observed that the velocity decreases with increasing Schmidt number. The velocity profiles for different values of magnetic parameter 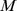 are shown in Fig. 5. The range of magnetic field is taken from 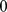 to 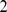. It is found that the velocity is decreasing with increasing values of 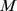 in both cases of ramped and isothermal plates. Physically, it is true due to the fact that increasing values of 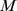 causes the frictional force to increase which tends to resist the fluid flow and thus reducing its velocity. It is further observed that when the magnetic field imposed on the flow is zero (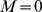), the MHD effect vanishes and the flow is termed as hydrodynamic flow.

Fig. 6 are plotted to see the difference between the ramped and isothermal plate velocities. The values of 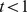 correspond to ramp velocity whereas 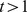 is for isothermal plate. It is found that ramp velocity is less than isothermal plate and converges faster. Further velocity in both cases increases with increasing time. The effects of inverse permeability parameter 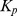 on the velocity profiles are presented in Fig. 7. It is found that velocity decreases with increasing 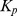 in both cases of ramp and isothermal plate. Physically, it is due to the fact that increasing permeability of the porous medium increases the resistance and consequently velocity decreases. This observation is an excellent agreement with the previous study [28]; Fig. 3. The effects of the shear stress 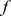 induced by the bounding plate on the non-dimensional velocity profiles are shown in Fig. 8. The velocity of fluid is found to decrease with increasing 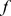 in both cases of ramped velocity and isothermal plate. Graphical results to show the influence of the effective Prandtl number 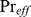 on velocity profiles are presented in Fig. 9. It is observed that the velocity is a decreasing function with respect to 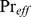. These graphical results are in accordance with [28]; Fig. 2.

The temperature variations against 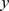 for various values of effective Prandtl number are highlighted in Fig. 10. The significant decrease of the temperature is found as a result of an increase of the effective Prandtl number. The fluid temperature decreases from maximum at the boundary to a minimum value as far from the plate in both cases of ramped and constant temperature. In Fig. 11 we have shown the temperature variations for two types of boundary conditions ramped and constant plate temperatures. It is noted that the fluid temperature is greater in the case of isothermal plate than in the case of ramped temperature at the plate. This should be expected since in the latter case, the heating of the fluid takes place more gradually than in the isothermal case [18]. Moreover, with increasing time, the temperature is found to increase in both cases of ramped and constant wall temperature. The concentration profiles for different values of Schmidt number 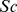, are shown in Fig. 12. It is clear from this figure that the concentration profiles and the concentration boundary layer thickness decrease with increasing values of 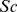. Physically, it is true, since increase of 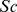 means decrease of molecular diffusivity which results in a decrease of concentration boundary layer. The concentration profiles for different values of time 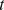 are presented in Fig. 13. It is observed that concentration profiles increase with increasing 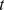.

## Conclusions

The purpose of this work was to analyze the unsteady MHD free convection flow of an incompressible viscous fluid over an infinite plate with ramped wall temperature and applies an arbitrary shear stress to the fluid. Exact solutions for velocity, temperature (for both cases of ramped and constant wall temperature) and concentration are obtained using the Laplace transform technique and expressed in terms of the complementary error function. They satisfy all imposed initial and boundary conditions. These solutions are plotted in various figures for different parameters of interest. It is found that velocity of the fluid 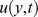 can be written as a sum of its mechanical and thermal components 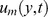, respectively 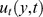. For the velocity solution in which the plate applies an oscillating shear stress to the fluid 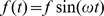, the mechanical part can be further written as a sum of the steady-state and transient solutions 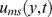, respectively 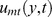. The thermal boundary layer thickness in case of ramped wall temperature is less than isothermal wall temperature. Magnetic parameter 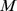 retards whereas the inverse permeability parameter 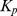 enhances the fluid motion. The thermal boundary layer, as well as the temperature of the fluid, increases in time and decreases with respect to the effective Prandtl number Pr*_eff_*. The concentration boundary layer thickness decreases with increasing values of 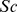 whereas increases with increasing 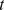.
